# Immunoserological Diagnosis of Human Borrelioses: Current Knowledge and Perspectives

**DOI:** 10.3389/fcimb.2020.00241

**Published:** 2020-05-19

**Authors:** Emilie Talagrand-Reboul, Alice Raffetin, Pierre Zachary, Benoît Jaulhac, Carole Eldin

**Affiliations:** ^1^UR 7290 Virulence Bactérienne Précoce, Université de Strasbourg, Centre Hospitalier Régional Universitaire de Strasbourg, Fédération de Médecine Translationnelle, Groupe Borréliose de Strasbourg, Strasbourg, France; ^2^National Reference Center for Borrelia, CHRU Strasbourg, Strasbourg, France; ^3^Department of Infectious Diseases, Centre Hospitalier Lucie-et-Raymond-Aubrac, Villeneuve-Saint-Georges, France; ^4^Aix Marseille Univ, IRD, SSA, VITROME, Marseille, France; ^5^IHU-Méditerranée Infection, Marseille, France

**Keywords:** borrelia (Borreliella) burgdorferi, lyme, relapsing fever Borrelia, serology, ELISA, CXCL-13

## Abstract

Spirochetes of the genus *Borrelia* are divided into relapsing fever borreliae and Lyme disease borreliae. Immunoserological assays have been poorly developed for relapsing fever borreliae, where direct detection methods are more adapted to the pathophysiology of these infections presenting with massive bacteraemia. However, emergence of the novel agent of relapsing fever *B. miyamotoi* has renewed interest in serology in this context. In Lyme disease, because direct detection methods show low sensitivity, serology plays a central role in the diagnostic strategy. This diagnostic strategy is based on a two-tier methodology involving a first test (ELISA) with high sensitivity and acceptable specificity and a second, more specific test (western blot) for diagnostic confirmation. The most frequent limitations and pitfalls of serology are cross reactions, false IgM positivity, a seronegative window period at the early time of the infection, and serologic scars with a suspicion of reinfection. International guidelines have thus been proposed to avoid these difficulties with interpretation. Finally, unconventional diagnostic tests have been developed recently in the context of a highly publicized disease, with widely varying results, some of which have no available evidence-based data. New two-tier testing strategies using two ELISA tests (C6 and WCS for example) to replace immunoblot are currently proposed by some authors and guidelines, and promising new tests such as CXCL-13 in CSF are promising tools for the improvement of the diagnosis of Lyme borreliosis.

## Introduction

Spirochetes of the genus *Borrelia* are widely distributed vector-borne pathogens. Within this genus, the borreliae have been classified based on phylogenetic differences related to ecological factors and clinical manifestations: relapsing fever species are mainly vectored by soft ticks (with the exception of the louse-borne *B. recurrentis* and *B. miyamotoi*, which is vectored by hard ticks) (Talagrand-Reboul et al., 2018) whereas Lyme disease species and relatives are transmitted by hard Ixodid ticks (Cutler et al., 2017), the latter species being known as *Borrelia burgdorferi* sensu lato complex. However, some authors advocate for the creation of a new *Borreliella* genus regrouping members of the Lyme disease group of borreliae, and this topic is still debated (Barbour et al., 2017; Margos et al., 2017).

Indeed, relapsing fever group and Lyme disease borreliae differ in many ways, and diagnostic methods, particularly regarding the place of immunoserological diagnosis, reflect these differences. In Lyme disease, following a localized infection (erythema migrans), bacteraemia is usually very moderate, of short duration, especially in Europe, and occurs at the very beginning of the dissemination that does not allow direct diagnosis from blood (Eldin et al., 2019a). But the seroreactivity to a spirochete isolated from *Ixodes* ticks in patients convalescing from Lyme disease was early reported by Burgdorfer et al. (1982). Subsequently, it enabled the development of the indirect diagnostic methods (i.e., serological assays) that are currently used for the biological diagnosis at the disseminated stage.

In contrast, relapsing fever borreliae can lead to massive bacteraemia during febrile episodes, which explains why the direct detection of the pathogen through microscopy, culture or PCR on a blood sample (Eldin et al., 2019a) is favored. In this context, specific serology tools have been poorly developed and are mainly used retrospectively following an acute episode.

Because public awareness of Lyme disease is currently high in Europe and in the USA, the reliability of diagnostic tests, particularly serology, is regularly questioned by a few physicians and some patient's associations, mainly through the internet and on social media, based on testimonies. Consequently, precise and timely reviews of current scientific data about the techniques and the rules of interpreting serologies are needed. In contrast, relapsing tick-borne borreliae, which represent a real public health problem in Africa and are also present in Europe, are poorly known by the populations of developed countries and are considered as neglected diseases (Fotso Fotso and Drancourt, 2015). However, the recent description of human cases of *Borrelia miyamotoi* in Europe (Platonov et al., 2011) and in the USA (Krause et al., 2013), transmitted by Ixodid ticks, has raised new interest in tick borne relapsing fever diagnostic tools, particularly serology. In this review, we report the current knowledge about immunoserological diagnosis of Lyme disease and relapsing fever borreliae and tools that are currently under development.

### Relapsing Fever Borreliae

Currently, the most accurate and useful diagnostic tools for the acute phase of relapsing fever are specific qPCRs and some multiplex qPCRs are also available (Eldin et al., 2019a). To date, no serological test is commercially available, and these techniques are currently performed for research purposes. Historically, Whole Cell Lysate (WCS) of *Borrelia hermsii* was used as the antigen source (Schwan et al., 1996), but early studies revealed that the antigenic variability of the different species of relapsing fever borreliae, and antigens shared with Lyme disease borreliae could cause both false positive and false negative results. Consequently, serological assays based on the GlpQ immunoreactive protein, which is absent from the Lyme disease borreliae, have been developed. This assay performed well in seroprevalence studies in the north-east of the USA, which were designed to investigate the prevalence of *B. miyamotoi* (Schwan et al., 1996; Krause et al., 2014). However, in this context, the GlpQ antigen may also react with other relapsing fever species found in the USA (*B. hermsii* for example) (Krause et al., 2018). These studies have also demonstrated that sera from patients with *B. miyamotoi* antibodies could also cross-react with ELISA and Western blot tests designed for the diagnosis of Lyme disease borreliae. Similarly, this phenomenon has been described for other relapsing fever species like *B. crocidurae* (Krause et al., 2014, 2018; Fotso Fotso and Drancourt, 2015).

A more recent study evaluated a GlpQ serological test in well-defined groups of patients: patients with PCR-confirmed *B. miyamotoi* infection, patients with Lyme borreliosis and patients with tick-borne encephalitis (Jahfari et al., 2017). This study found a global sensitivity of 69% and a specificity of 98 and 92% for IgM and IgG assays, respectively (Jahfari et al., 2017). A more recent study performed in the Netherlands assessed the values of the association of two assays using GlpQ and four antigenic Variable Major Proteins (Vmps) and showed that several combinations of GlpQ and Vmps increased the sensitivity and/or specificity compared to the use of single antigens (Koetsveld et al., 2018).

Regarding other immunological based techniques, monoclonal antibodies have been developed for the specific detection of *B. crocidurae* by immunofluorescence assay (Fotso Fotso et al., 2016). The aim of this study was to provide a test which was well-suited to rapid point-of-care treatment in tropical areas with no specialized laboratory, but further studies are needed to assess its feasibility in real-life conditions.

## *Borrelia burgdorferi* Sensu Lato

### Serologic Tests and Diagnostic Accuracy

Due to the different limitations of direct detection (culture, PCR) in terms of timescale, technical complexity and sensitivity (Waddell et al., 2016), and contrary to relapsing fever borrelioses, the diagnosis of Lyme borreliosis is currently based primarily on serology, which is intended to confirm or infirm whether the patient's immune system has been in contact with *B. burgdorferi* sl. In practice, the two-tier methodology is currently recommended in most countries in national and international guidelines for the serodiagnosis of Lyme borreliosis, both in Europe and in America (Eldin et al., 2019b). This biological diagnostic strategy aims to improve the performance of laboratory tests by combining a highly sensitive test at the first stage with, for positive or equivocal results, a confirmatory highly specific test. The first-step serology is currently mainly performed by ELISA (Enzyme Linked ImmunoAssay) or sometimes by IFA (Indirect ImmunoFluorescence Assay) test. The second test corresponds to an immunological fingerprint method (Western-blot, line-blot or dot-blot) that can confirm or infirm the first test and also give a typing of the immune response of the patient based on the nature of the immunodominant antigen reacting with the patient antibodies.

### First Step Serology Tests

#### ELISA Tests or Equivalents

The first-step serology is currently most often performed by an ELISA test. Schematically, the *Borrelia* antigens (Ag) are coated in wells. If there are antibodies (Ab) against *B. burgdorferi* sl in the human serum sample, they will form Ag-Ab complexes. The complexes are then fixed with a conjugate of anti-human IgG or IgM antibodies coupled with an enzyme (e.g., horseradish peroxidase). The complexes are revealed by the addition of a colorimetric substrate (e.g., tetramethylbenzidine). Finally, the enzymatic reaction is chemically stopped, and the optical density of the well is measured using a spectrophotometer. In addition to ELISA tests, other equivalent techniques can be used as a first-step: ELFA (Enzyme Linked Fluorescent Assay), CLIA (ChemiLuminescence ImmunoAssay) and MMIA (Multiplexed Microbead ImmunoAssay). In the CLIA technique, the enzyme converts a substrate (e.g., luminol) into a chemiluminescent signal that is measured by a photomultiplier in Relative Light Units (RLU), and the Ag may be coated on paramagnetic particles that act as a solid phase instead of a well in the microplate (Ledue et al., 2008). In the most recent MMIA technology, polystyrene microspheres (or microbeads), also acting as a solid phase, are coated with the *Borrelia* antigens to detect specific Ab. This method may be adapted to different types of conjugate and allow the separate detection of two Ab isotypes in a single well. The signal is measured by fluorescent microsphere counting using a cytometer (Reslova et al., 2017).

The antigenic preparations used in the different ELISA or equivalent assays correspond to: (1) whole cell sonicates of *B. burgdorferi* culture, (2) purified native antigens (whole or selected antigens), (3) recombinant antigens, such as OspA, OspC, BmpA, DbpA, p41, VlsE proteins (Lawrenz et al., 1999), or (4) synthetic peptides, including “C6,” a region of VlsE which is well-conserved between strains (Liang et al., 1999) and “pepC10” which corresponds to the C-terminal 10-amino-acid residues of OspC (Mathiesen et al., 1998). Some tests use a mix of different antigens (e.g., whole antigens and recombinant VlsE) which may improve the sensitivity and/or specificity of the assay (Marangoni et al., 2008). When using whole cell sonicates of *Borrelia*, immunocapture-based tests, where the solid phase is coated with μ chain-specific anti-human IgM Ab, are of interest for the early detection of IgM, since fewer cross-reactions with unspecific antibodies have been reported (Hansen et al., 1991).

The diversity of *Borrelia* species involved in Lyme borreliosis in Europe increases the complexity of understanding this serodiagnosis. This polymorphism seems to have little influence on the results when obtained with ELISA tests using whole cell antigens. On the other hand, this polymorphism appears to reduce the overall sensitivity of tests based on recombinant antigens, which require the use of a mixture of recombinant antigens of the three main pathogenic species of *Borrelia* for better performance (Hauser et al., 1998). Therefore, the vast majority of the current ELISA kits or equivalents available in Europe use a mixture of antigens from the three major pathogenic species in Europe: *B. afzelii, B. garinii*, and *B. burgdorferi* ss.

The sensitivity of commercial ELISA tests on sera has been estimated at 54% (CI_95%_ = 44–64%) at the localized stage (erythema migrans), 81% (CI_95%_ = 70–89%) in cases of neuroborreliosis, 96% (93–100%) in Lyme arthritis and 97% (CI_95%_ = 94–99%) in acrodermatitis chronica atrophicans (ACA) in a European meta-analysis (Leeflang et al., 2016). In the same study, IgMs have a much lower sensitivity than IgG in ACA and arthritis, whereas IgGs have a lower sensitivity at the localized stage (Leeflang et al., 2016). The accuracy of ELISA tests were similar in an American meta-analysis with sensitivity at 54% (CI_95%_ = 43–65%) in erythema migrans, 79% (CI_95%_ = 66–88%) at the early disseminated stage and 95% (CI_95%_ = 86–98%) at the late disseminated stage (Waddell et al., 2016). The specificity of the commercial ELISA assays is generally estimated at between 90 and 97% in healthy controls (Leeflang et al., 2016; Waddell et al., 2016).

Overall, ELISA tests or equivalents allow an objective reading of the test with a quantitative signal, a secured automated process from primary samples and an overall high sensitivity at the disseminated stage of the disease.

#### IFA Tests

In the indirect IFA methodology, the *Borrelia* antigen is coated on a slide well. After incubation with human serum and wash, a fluorescein labeled anti-human globulin Ab is added. Finally, detection is performed using fluorescence microscopy. The antigens used are *Borrelia* smears alone or in combination with immunodominant antigen spots (e.g., Vlse, OspC). The IFA tests are much less often used than ELISA in the first-step serology because they are not automatable, the reading is subjective and the inter-laboratory reproducibility is lower (Hunfeld et al., 2002).

### Second Step Serology Tests

#### Immunoblot Tests

In the current Lyme borreliosis serodiagnosis, positive or equivocal human serum using ELISA or IFA should be tested with a second-step test such as immunoblot assays (two-tier methodology). In immunoblots (IB), the antibody response is evaluated against the different antigens which are separated and fixed on a solid support, generally nitrocellulose strips. Anti-human IgG or IgM Ab conjugated to an enzyme are used to bind the Ag/Ab complexes and detection is performed by the addition of a chromogenic substrate (Dressler et al., 1993). The detection is visual or automated, with the latter being less subjective. In commercial tests, *Borrelia* antigens correspond either to whole cell antigens with proteins separated by electrophoresis according to their molecular weight (Western-blot assays), to purified proteins (line blot), recombinant proteins (spotted on a membrane in dot blot format), or a mixture of native and recombinant proteins.

Different guidelines have been proposed for traditional Western-blot assays (WB) using, for example, in the United States the Bbss 297 strain isolated from a patient with neuroborreliosis (US, Connecticut) and corresponding to the CDC criteria [Dressler et al., 1993; Centers for Disease Control and Prevention (CDC), 1995; Engstrom et al., 1995] or, in Europe, using the *B. afzelii* Pko strain isolated from a German erythema migrans (Hauser et al., 1999). IgM WB should be considered positive if at least two of the following bands are present: p24 (OspC), p39 (BmpA) and p41 (flagellin) using the American strain 297, or at least one of these bands (strong p41 band) and p17 (DbpA), using the European strain Pko. IgG WB should be considered positive if at least five bands are present from p18, p21 (OspC), p28, p30, p39, p41, p45, p58 (not GroEL), p66, and p93 using the strain 297 or at least two bands from p14, p17, p21, OspC, p30, p39, p43, p58, and p83/100 using the strain Pko. Unspecific reactions are frequent with the flagellin antigen (p41) (Dressler et al., 1993). Line blots make it easier to interpret the results of these tests than WB (Hunfeld and Kraiczy, 2009). Line blots based on the use of recombinant *Borrelia* antigens have been associated with an improvement in sensitivity without loss of specificity in the early disseminated stage, adding recombinant VlsE and DbpA proteins (Schulte-Spechtel et al., 2003). Thereby, a new interpretation criterion of IB has been proposed, which considers a test to be positive when the VlsE band is detected, with a significant improvement in the disseminated early stage diagnosis, that may replace IgM IB testing (Branda et al., 2010). In neuroborreliosis from two European countries, the IgG seroreactivity of VlsE alone surpassed that of other antigens commonly used (p100, p58, p39, OspA, OspC, and p18) compared to control patients (Dessau et al., 2015). IB have been recently miniaturized in a microarray format with probable equivalent performances to other commercial immunoblots (Theel et al., 2018). Given the diversity of genospecies involved in Lyme borreliosis in Europe compared to the US, almost all of the available IB kits use combinations of recombinant antigens from strains belonging to different species, generally the three main European pathogenic species (*B. afzelii, B. garinii, B. burgdorferi* ss) possibly associated with *B. bavariensis* and *B. spielmanii*, also responsible for some cases of Lyme borreliosis. Considering the intrinsic performance features of IB alone, European and American meta-analyses have shown that IB tests are not more sensitive than ELISA tests as a whole, either at the localized stage or in case of Lyme arthritis or neurological manifestations. In particular, the mean sensitivity of IB ranges from 52% (in-house) to 61% (commercial) at the localized stage, and from 69% (in-house) to 81% (commercial) in Lyme neuroborreliosis (Leeflang et al., 2016; Waddell et al., 2016). The specificity of IB assays is also variable and generally ranges from 86 to 97% (Leeflang et al., 2016; Waddell et al., 2016).

Overall, the IB tests require a highly technical skill for the preparation of the antigen and the strips, for the incubation and washing steps, and for the reading of the result. However, the criticality of this technical expertise tends to decrease due to commercial technical developments (commercial tests, lineblots, incubation and washing automation, automatic pipetting, automatic reading of the result). Even if the technical modernization enables the practice of assays with a good level of reliability, IB tests or further new immunotyping tests would require a better standardization for the type of antigen and for interpretation (i.e., both selected by scientific consensus rather than scoring algorithms chosen by the manufacturers) in order to enhance the diagnostic performances.

#### Biological Sample for Serology Testing

The search for antibodies is carried out in blood (serum and/or plasma) and is of interest at the different stage of disease except at the erythema migrans stage, and in CSF in the event of suspected neuroborreliosis. There is no interest in carrying out this serodiagnosis in the synovial fluid, which is firstly a biological sample that appears not more sensitive than serum in case of Lyme arthritis (Eiffert et al., 1998) and secondly is highly permeable to proteins, leading to synovial IgG levels reaching serum IgG levels even in the absence of Lyme arthritis (Strle and Stanek, 2009).

The confirmation of Ab specificity by immunoblot technique is required in the blood (two-tier testing) but it is not recommended in CSF because it is less standardized than in the serum and requires a large amount of CSF. Identification of intrathecal specific antibody synthesis in the biological confirmation of neuroborreliosis is more valuable for the biological diagnosis (Reiber and Peter, 2001; Stanek et al., 2011; Dessau et al., 2018).

### Intrathecal Synthesis of Specific Ig

When neuroborreliosis is suspected, the biological investigation should not be limited to blood serology because its positivity alone is not sufficient to establish the diagnosis of neuroborreliosis. Moreover, at the beginning of an acute neuroborreliosis (generally ≤ 6 weeks, not > 3 months), IgG serology may be negative in the serum and positive only in the CSF (Hansen and Lebech, 1991, 1992; Tumani et al., 1995; Ogrinc et al., 2013). For example, an early large Danish cohort of 187 definite cases of neuroborreliosis has recorded 44 patients (24%) that had negative IgG serology in serum after a median duration of 19 days after disease onset (6–54 days), including 30/44 patients with isolated positive IgM (Hansen and Lebech, 1992).

In the case of a positive blood serology, the presence of intrathecal synthesis of anti-*Borrelia* immunoglobulins is an important biological argument for the diagnosis of neuroborreliosis (Wilske et al., 1986; Hansen and Lebech, 1991; Tumani et al., 1995; Blanc et al., 2007). Thereby, in the presence of a neurological clinical picture and a positive blood serology, it is necessary to look for a specific intrathecal synthesis of anti-*Borrelia* antibodies. In addition, a lumbar puncture will also make it possible to identify a predominant lymphocytic pleocytosis that is common very early in neuroborreliosis (other than peripheral neuropathy), thus constituting an important additional diagnostic element (Tumani et al., 1995; Ogrinc et al., 2013).

The production of CSF antibodies occurs within 2–6 weeks of the onset of disease (Hansen and Lebech, 1991). While the specificity of this intrathecal synthesis test is excellent, its sensitivity generally ranges from 70 to 90%, which is less sensitive at the very beginning of neuroborreliosis (Ogrinc et al., 2016). Indeed, the seroconversion in CSF may appear after the medical management of suspected neuroborreliosis. This was observed in 11 (6%) patients of the Danish neuroborreliosis cohort for which the initial serology in CSF was negative for both IgG and IgM after 10 days of disease onset in median (4–30 days) (Hansen and Lebech, 1992). The date of the onset of neurological signs should therefore be considered when interpreting the results of CSF serology.

In contrast, a positive serology in the CSF alone does not systematically link a neurological picture to a *Borrelia* infection. The principle of the intrathecal synthesis research is to compare the ratio of anti-*Borrelia* antibody levels in CSF and serum to the level of total albumin or immunoglobulins G in the CSF and serum. When the Reiber's diagram prerequisites are fulfilled, the formula used is: Antibody index = [CSF specific IgG rate (U/mL)/serum specific IgG rate (U/mL)]/(CSF total IgG quantity (mg/L)/serum total IgG quantity (mg/L)]. A CSF/serum Antibody index ≥ 1.5–2 indicates a positive intrathecal synthesis of anti-*Borrelia* antibodies (Reiber and Peter, 2001). This enables the distinction between a passive transudation of serum antibodies through the blood-brain barrier from an intrathecal production of anti-*Borrelia* antibodies that signals neuroborreliosis.

The positive predictive value of isolated IgM in CSF is insufficient to confirm the clinical suspicion of early neuroborreliosis, and a serological control (CSF and blood) 6 weeks later gives a higher level of evidence (Pierer et al., 1999). Importantly, a haemorrhagic CSF is not acceptable for this serological analysis, as the presence of blood-borne antibodies distorts interpretation of the results.

### Kinetic of the Humoral Immune Response

In general, the number of *Borrelia* proteins recognized by the immune system increases with the duration of the disease. The initial immune response to *Borrelia* is primarily directed against OspC, BbK32, and flagellin, proteins that are early *in-vivo* expressed proteins (Aguero-Rosenfeld et al., 1993; Fikrig et al., 1997) (Table 1). OspC protects *Borrelia* from destruction by phagocytosis and BbK32 inhibits the classical pathway of the complement. These proteins are therefore required at an early stage to establish human infection (Carrasco et al., 2015; Garcia et al., 2016).

**Table 1:** Variations in the *Borrelia* immunodominant antigen expression during human infection (from Aguero-Rosenfeld et al., 1993, 1996; Dressler et al., 1993; Engstrom et al., 1995; Fikrig et al., 1997; Hauser et al., 1997; Akin et al., 1999; Panelius et al., 2003).

**Early antigens**	**Early/Late antigens**	**Late antigens**
OspC (p21-p25, Major outer surface lipoprotein C) BbK32 (Fibronectin-binding protein) Flagellin (p41)	VlsE (vmp-like sequence E) Dbpa (p17-p18, Decorin-binding protein A) OppA-2 (p58, Oligopeptide-binding protein) BmpA (p39) p14 p28 p43 p45	OspA (p31) p30 p66 p83/100 p93

Globally, the highest IgM rates are generally observed at the EM stage and the highest IgG rates at arthritis or ACA stage (Aguero-Rosenfeld et al., 1996; Stanek et al., 2011; Lenormand et al., 2016) (Table 1). At the initial stage of EM (localized infection), only 50% of patients develop IgM, which occurs within 2–4 weeks of the onset of disease, particularly if the patient shows signs of dissemination (myalgias, arthralgia) (Strle and Stanek, 2009). IgM synthesis peaks at 6–8 weeks and usually decreases gradually after 3 months but may take more than a year or even a decade to disappear, even after effective treatment (Feder et al., 1992; Engstrom et al., 1995; Aguero-Rosenfeld et al., 1996; Kalish et al., 2001). IgM synthesis is followed by an IgG response to many proteins, initially against VlsE, OspC, BbK32, and flagellin, then against DbpA, BmpA and p58 proteins (Aguero-Rosenfeld et al., 1996; Hauser et al., 1997; Panelius et al., 2003) (Table 1).

In the late disseminated stage of Lyme borreliosis, the IgM is infrequently positive. For example, only four IgM and IgG positive serologies were found in a cohort of 20 ACA cases confirmed by histology, culture and/or PCR (Lenormand et al., 2016). Conversely, IgG response is massive due to a very large number of *Borrelia* antigens in late disseminated borreliosis, sometimes including the OspA protein (Dressler et al., 1993; Akin et al., 1999) (Table 1).

### Limitations of Lyme Borreliosis Serodiagnosis

#### Cross Reactions

Cross-reactions between Lyme borreliosis serological tests and other spirochetes (*Borrelia* relapsing fever agents, *Treponema pallidum, Leptospira interrogans* and oral treponemes in subjects with gingivitis or periodontal diseases and *Borrelia* relapsing fever agents) have been reported, as well as with auto-immune or inflammatory pathologies (anti-nuclear antibodies, rheumatoid factor), bacterial endocarditis agents, other tick-borne disease agents (*Anaplasma phagocytophilum, Ehrlichia chaffeensis*) or viral infections (Ebstein-Barr virus, B19 Parvovirus, HIV) (Magnarelli et al., 1987, 1990; Raoult et al., 1989; Aguero-Rosenfeld et al., 1993; Kaell et al., 1993; Keymeulen et al., 1993; Engstrom et al., 1995; Wormser et al., 1996; Wong et al., 1997; Tuuminen et al., 2011).

Molins et al. have compared the reactivity against 144 sera from potentially reactive diseases for three types of EIA tests (Molins et al., 2017). The cross reaction rates were higher for a WCS-based ELISA (27%) and for a DbpA-OspC IgM (15%) recombinant EIA test than for a VlsE-DbpA-OspC IgG (3%) and a C6 total Ig (5%) recombinant EIA test. For the WCS-based and recombinant IgM EIA tests, the highest rate of cross-reactions were observed in syphilis (*n* = 20), rheumatoid arthritis (*n* = 21) and infectious mononucleosis (*n* = 30) with false positivity rates of 85/20%, 53/27%, and 10/19%, respectively. In a comparative study of two EIA tests (i.e., one recombinant CLIA and one purified native antigen+rVlsE ELISA; Marangoni et al., 2008), there was also a higher rate of false positive or equivocal results in the IgM isotype than in the IgG one, from 5% (1/22) in syphilis to 27% (3/11) in infectious mononucleosis for both tests. In the IgG tests, the cross-reactions involved 2 and 6 of the 100 tested samples in ELISA and CLIA tests, respectively. Finally, the IgM EIA tests lead to a high rate of false positives in cross-reactive diseases regardless of the type of assay. In ELISA tests or equivalents, the frequency of these cross reactions may be reduced by prior adsorption of the sera to be tested on a suspension of treponemas or Gram-negative bacilli, although sometimes with reduced sensitivity, or by using immunocapture-based methods especially for IgM (Hansen et al., 1991). Even if the frequency of cross-reactions seems low in some commercial WCS-based ELISA tests as in the newer recombinant tests, the knowledge of the false-positive rate is necessary for contextualized interpretation of results. Sera from patients with often cross-reactive diseases can be used to evaluate the specificity of diagnosis tests in such contexts, that is regularly specified for commercial tests.

Immunoblots do not harbor a complete immunologic pattern and the interpretation criteria by the number and type of positive bands generally makes it possible to reject false positive cases (Raoult et al., 1989; Hauser et al., 1999).

#### Seronegative Window Period (Serological Silence)

As for all adaptive immune responses to microbiological agents, there is a physiological delay between the infecting tick bite and the time when the specific antibodies production reaches a detectable rate. After an infecting tick bite, erythema migrans appears generally between 2 and 30 days and seroconversion occurs after 2–4 weeks (Aguero-Rosenfeld et al., 1993, 1996). Therefore, a non-compressible seronegative window period (serological silence) must be considered, where the patient may present early Lyme disease, while serodiagnosis tests are negative. Moreover, the sensitivity of serodiagnosis tests in the early cutaneous stage (around 50%) does not rely on the intrinsic qualities of tests alone, but mainly on the variable level of seroconversion at this early local stage of the disease, and possibly to a clinical misdiagnosis of EM (e.g., skin reactions due to tick-bite or insect-bite) (Aguero-Rosenfeld et al., 1993, 1996). A recent meta-analysis showed that LB serological tests presented heterogeneous sensitivity, depending on the stage of the disease: 50% (95% CI = 40–61%) for localized EM, 77% (95% CI = 67–85%) for Lyme neuroborreliosis, (Leeflang et al., 2016).

Therefore, a negative serology at an early stage of the disease does not necessarily exclude Lyme borreliosis. The serological test might be repeated 3 weeks later and demonstrate seroconversion. For erythema migrans, the serology is not useful as this skin lesion is pathognomonic.

#### Background Seropositivity and Previous Contact Without Disease

A positive serology does not necessarily imply an active infection and may result from previous exposure to *B. burgdorferi* sl, as specific IgM and IgG can remain several years after the initial infecting bite (Kalish et al., 2001). Therefore, serological tests must not be used in the post-treatment follow-up. In large cohorts of healthy patients (i.e., seroprevalence surveys), seropositivity may indicate their exposure level but not the rate of Lyme borreliosis (Rigaud et al., 2016). Fahrer et al. performed a longitudinal study in Switzerland between 1986 and 1993 to study the infection rate following a tick bite. Three hundred and five patients presented a seroconversion with a positive IgG serological test and patients had no initial clinical signs. Of these 305 infected patients with *Borrelia*, more than 95% were still asymptomatic after a 7-year follow-up (Fahrer et al., 1998). Therefore, positive serology should not always result in an antibiotic therapy that would be inefficient, unnecessary and even sometimes dangerous due to possible adverse events. Clinical signs and symptoms should prevail over serological testing which should not be used as a screening test for Lyme borreliosis but as a part of the diagnosis strategy together with clinical and epidemiological data.

#### Reinfection

When Lyme borreliosis is correctly treated, there is no relapse, but reinfections after new infecting tick bites are possible (Nadelman et al., 2012). Because of the previously discussed blood persistence of antibodies, the serodiagnosis of patients with possible reinfection is a major problem for clinicians. Outside the early localized stage (erythema migrans), a serological analysis is recommended, but the results should be interpreted with caution. In such cases, it would be informative to perform both acute and convalescent serological tests to detect any increase in ELISA titers or modification in the seroreactivity pattern by immunoblot (Pfister et al., 1986).

#### Positive Predictive Values of Tests

In an endemic region where seroprevalence is supposed to be around 5%, the expected prevalence of Lyme borreliosis when a serological test is prescribed may be at a maximum of 1% in a group of patients with unspecific symptoms and of 10% in a group with an accurate clinical setting (excluding EM). Using a serological EIA test (using both IgG and IgM antibodies) with a specificity of 95% and a sensitivity of 95%, the probability of a negative result for someone suffering from disseminated Lyme borreliosis would be low, at only 0.05 and 0.58%, respectively (i.e., 1- negative predictive value). At the same time, in positive tests, due to the specificity of 95%, only 16% of the seropositive persons tested with unspecific symptoms would actually have Lyme borreliosis (i.e., positive predictive value), and 68% in the group with more accurate symptoms. The two-tier testing strategy aims to enhance this positive predictive value by increasing the specificity of the serological testing (Johnson et al., 1996; Wilske, 2002), and, in order to increase the pre-test value, a serological test must only be requested in cases of typical clinical pictures and not regarding unspecific disorders such as fatigue or myalgia (Dessau et al., 2018).

#### Poor Specificity of IgM

With the exception of a few tests that search for total antibodies, IgM is currently routinely searched for using a separate test from IgG. IgM can be detected alone or concomitantly with the presence of IgG. Their presence may correspond to a recent infection, as in the initial phase of the disease, but it is also possible to detect residual levels of IgM in late manifestations such as Lyme arthritis or ACA (Lenormand et al., 2016; Grillon et al., 2019). Their presence is therefore not synonymous with a recent infection and correct interpretation will depend strongly on the clinical context and the notion of a recent tick bite.

Moreover, careful attention should be paid to the biological interpretation of isolated positive IgM results, since this does not necessarily reflect an active infection. In cases where the clinical picture raises suspicions of disseminated manifestations of Lyme disease, an isolated positive IgM result should be regarded mostly as a cross-reaction result and not as biological proof of a *Borrelia* infection. In the overwhelming majority of cases where a serological follow-up was possible, no IgM-IgG seroconversion was observed; as exemplified in a large cohort of professional football players in Germany where 2.3% of positive IgM athletes with neither clinical Lyme disease nor seroconversion was observed in the follow-up (Breitbart et al., 2019). As a consequence, isolated positive IgM for specimens collected more than 6 weeks after the onset of the symptoms should be primarily considered as a “false positive.” The CDC guidelines have recommended to not rely on the IgM testing after 30 days from the onset of the disease [Centers for Disease Control and Prevention (CDC), 1995; Engstrom et al., 1995]. More recently, Seriburi et al. demonstrated in a retrospective study of patients consulting an Infectious Diseases physician for a suspicion of Lyme borreliosis, that 50 of 182 patients (27.5%, CI_95%_ = 21.1–34.6) had a false positive IgM immunoblot, and that 78.0% of them had received unnecessary antibiotics (Seriburi et al., 2012).

Webber found similar results in their recent retrospective study of all Lyme borreliosis serological tests ordered at US Air Force healthcare facilities in the United States (January 2013–December 2017). They found that 18,410 sera had been tested (17,058 immunoassays; 1,352 immunoblots) from 15,928 individuals. Of the 1,352 IgM immunoblots, 249 (18.4%) were positive and 212 cases were assessed. Repeated serological tests, insufficiently documented cases, and patients with a past medical history of Lyme borreliosis were excluded. Of the 212 cases, 113 (53.3%) were considered as false positives and 91/113 (80.5%) received an unnecessary antibiotic therapy (Webber et al., 2019). As a consequence, four criteria should be systematically sought when an IgM test for *Borrelia* is found positive without IgG: (i) verification of the positivity criteria for serology; (ii) high probability of tick exposure (depending on the geographic area and of the season); (iii) symptoms and clinical signs highly evocative of early Lyme borreliosis; (iv) IgG seroconversion on retesting more than 4 weeks later.

#### False Negative Serology?

As mentioned above, serological testing for Lyme borreliosis performs satisfactorily. Serological tests are not negative in disseminated Lyme borreliosis except at the very beginning of early neuroborreliosis or, in rare cases for deeply immunocompromised patients. Only two well-described cases of seronegative Lyme borreliosis have been identified in particular contexts: (i) one case of Lyme arthritis in a patient who was receiving glucocorticoid injections for an idiopathic juvenile arthritis diagnosed 5 years previously; (ii) and one case of neuroborreliosis in a patient receiving treatment for chronic lymphatic leukemia (Harrer et al., 2007; Holl-Wieden et al., 2007). Two other cases of acrodermatitis chronica atrophicans with negative serology and no known immunodepression have also been described (Berger et al., 2003) but none other similar cases were published by others.

Moreover, a second reason is sometimes evoked as a hypothesis and applies in the case of patients who have received an antibiotic therapy at the very early stage that might have stopped the seroconversion (e.g., prophylactic antibiotics after a tick bite, or a prescribed antibiotic therapy for an intercurrent bacterial infection, etc.) (Aguero-Rosenfeld et al., 1996; Aucott et al., 2009). Aucott et al. demonstrated that of 25 patients presenting with an acute viral-like illness a few days or weeks after a tick bite, and with negative serology for Lyme borreliosis even after a second control, 70% had received prophylactic antibiotics. This group of 25 patients was compared to a group of 7 patients who had not received any antibiotics after a tick bite and who presented an acute viral-like illness a few days or weeks afterwards with a positive serology for early Lyme borreliosis or an objective seroconversion. Nonetheless, there are several bias to this study that do not allow any conclusion: the very small number of patients in the seronegative group, the very small number of patients in the control group, and most of all, the absence of a clear clinical definition of a proven early Lyme borreliosis. Other authors have stressed the fact that most patients receiving early effective treatment for culture-confirmed erythema migrans still seroconverted, as observed in an American cohort of 47 patients who had serological testing of acute and convalescent phases (Nowakowski et al., 2001; Halperin et al., 2013).

### Indications of Serology and International Rules of Interpretation

Most of the recent European guidelines recommend a two-tier test strategy: ELISA first, and in case of a positive result a western-blot (or Immunoblot, line blot, dot blot) to confirm or infirm the positivity. Except in the case of EM, when serology is negative and that there is a strong clinical suspicion, the serological test should be repeated 3 weeks later. In the case of a disease lasting within 6–8 weeks or more, a negative serological assay enables the ruling out of disseminated Lyme borreliosis as diagnosis (Dessau et al., 2018).

Because of the characteristics of the tests, detailed above, the first test should be carried out in suspected cases only, but not as a screening test in healthy subjects or in patients with unspecific signs, to avoid misleading interpretations (Dessau et al., 2018). Confirmation of IgG and IgM antibody positivity using the second line test is then required to increase the positive predictive value of serological assays.

A serological test for Lyme borreliosis is indicated when the patient has been exposed to tick bites and when the clinical features evoke a disseminated Lyme borreliosis infection such as lymphocytoma, multiple erythema migrans, meningo-radiculitis, arthritis, conduction block or cardiac rhythm impairment, uveitis, acrodermatitis chronica atrophicans, encephalomyelitis etc. (Eldin et al., 2019b). Serology is not recommended: (i) for screening, as antibodies only reflect an exposure to *Borrelia* and not the disease itself, (ii) for asymptomatic patients following a tick bite, as asymptomatic seroconversion is possible, in which case patients do not need a treatment, (iii) for the follow-up of patients with Lyme borreliosis once they have completed a well-conducted treatment, as the serology can remain positive for years, (iv) for erythema migrans, as the serology can still be negative at this early stage and the skin lesion is pathognomonic.

### Performance of Two-Tier Methodology

In Europe, the sensitivity of the two-tier methodology was estimated to be 55% (CI_95%_ = 32–77%) at the erythema migrans stage, 87% in Lyme neuroborreliosis -considering serum results only- (CI_95%_ = 60–98%), 93% in Lyme arthritis (CI_95%_ = 68–100%) and 100% in ACA (CI_95%_ = 77–100%) (Branda et al., 2013). A meta-analysis of American studies revealed similar data regarding the accuracy of the two-tier testing algorithm, which increased with the duration of the disease from a median sensitivity of 46% (CI_95%_ = 39–54%) in the localized cutaneous stage, to 90% (CI_95%_ = 78–95%) at the early disseminated stage and 99% (CI_95%_ = 96–100%) at the late disseminated stage (Waddell et al., 2016). The specificity of the two-tier methodology is very high, reaching ≥ 99%, which significantly increases the predictive positive value of tests (Branda et al., 2013; Leeflang et al., 2016; Waddell et al., 2016).

Regarding the rules of interpretation of the serology according to the clinical context, the following principles are uniformly found in international guidelines (Eldin et al., 2019b): (i) a serological test with isolated positive IgM more than 6 weeks after a tick bite is considered as a false positive, (ii) at a late disseminated stage, the absence of IgG for *Borrelia* should encourage a differential diagnosis and exceptional causes of false negative serology, (iii) high levels of antibodies against *Borrelia* observed years after a well-conducted treatment should not result in prescription of a second line of antibiotics. Serological follow-up is not recommended in these cases, and the post-treatment follow-up is based on clinical outcome. In Lyme neuroborreliosis, all the recent guidelines recommend simultaneously sampling blood and cerebrospinal fluid (CSF), performing a serological test in both serum and CSF, and determining the specific antibody index for *Borrelia* in CSF (Eldin et al., 2019b; Jaulhac et al., 2019). In the case of a positive serology with no evocative clinical signs, no antibiotic treatment is required (Eldin et al., 2019b; Jaulhac et al., 2019).

### “Unconventional” Diagnostic Tests

In addition to serological tests, other techniques have been suggested to improve the diagnosis of Lyme disease. A recent systematic review included 40 studies of unconventional tests (Raffetin et al., 2019). The QUADAS-2 quality assessment was used for each study, revealing a high risk of bias in 25/40 studies and uncertainty regarding applicability in 32/40. Three kinds of tests were identified: tests exploring inflammatory and auto-immune responses (CXCL-13, CCL-19, Apolipoprotein B-100); tests exploring cellular immunity (Lymphocyte transformation test, IGN-gamma ELISPOT, IFN-alpha, CD57+ NK-cells); and direct microbiological tests (xenodiagnoses, microscopy, OspA membrane protein detection, and rapid diagnostic tests). Some tests could not be included in this analysis since the princeps study did not specify their performances, including CCL-19 (Aucott et al., 2016), Apolipoprotein B-100 (Crowley et al., 2015; Strle et al., 2017), IFN-alpha (Jacek et al., 2013), CD57+ NK-cells (Marques et al., 2009), OspA membrane protein (Cheung et al., 2015; Magni et al., 2015) and xenodiagnosis (Marques et al., 2014).

**Figure 1:**
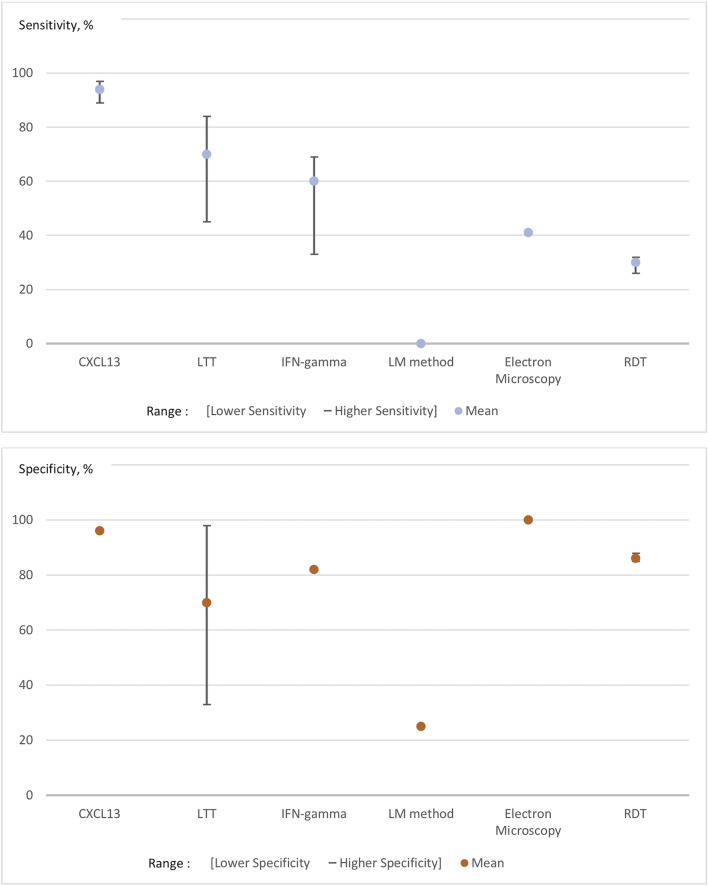
Pooled sensitivities and specificities of unconventional tests for Lyme borreliosis. The lower/higher Se/Sp represent the range of the sensitivities and specificities that we found in the studies included in this review of the literature. The numbers in the abscise are representing the percentage of sensitivities and specificities. LTT, Lymphocyte Transformation test; LM method, Light Microscopy Method; RDT, Rapid Diagnostic Test.

Figure 1 presents the range of the sensitivities and specificities (such as mentioned in each of the studies included in this review of the literature) of unconventional diagnostic tests (CXCL-13, lymphocyte transformation test, IFN-gamma, Electron Microscopy, LM-method for microscopy, and rapid diagnostic tests) (Raffetin et al., 2019). As illustrated in Figure 1, CSF concentration of C-X-C motif chemokine Ligand-13 (CXCL-13), which is a molecule produced by antigen-presenting cells to attract B lymphocytes, is the only test that can bring significant improvement. Unconventional LB-diagnostic tests could be classified as follows in this systematic review: accurate diagnostic tests, which need a better standardization and a better cut-off definition, such as CXCL-13 in cerebrospinal fluid; promising tests still under clinical evaluation and not used routinely, such as OspA membrane protein detection, CCL-19, and IFN-alpha; uncertain tests, because of a lack of proof of their performance in the studies (controversial results, poor methodological quality), including lymphocyte transformation tests and IFN-gamma ELISPOT; non-validated tests with too low sensitivity and/or specificity, such as CD57+ NK-cells and rapid diagnosis tests; tests for research purposes as they could be accurate but could not be practically developed in routine practice, including xenodiagnosis and microscopy (Raffetin et al., 2019). In this study, no test performed acceptably for late disseminated Lyme disease. Other diagnostic tests have been publicized, mainly via in the internet, but their sensitivities, specificities and reproducibility are heterogeneous and/or unassessed and, as a result, they should not be used.

### New Tools and Perspectives

#### CXCL-13 in CSF

As mentioned above, CSF CXCL-13 is one of the most promising tools currently in development. Several recent studies have shown that CSF CXCL-13 concentrations were high in patients with Lyme neuroborreliosis (two meta-analyses) (Yang et al., 2017; Rupprecht et al., 2018), in one retrospective controlled study (Markowicz et al., 2018) and one prospective uncontrolled study (Pietikäinen et al., 2018). Also, a previous study has shown that levels of CXCL-13 were detectable before antibodies in cerebrospinal fluid and that it fell shortly after antibiotic treatment (Rupprecht et al., 2005). CSF CXCL-13 might, therefore, be of potential interest as a therapeutic marker for follow-up (Senel et al., 2010; Yang et al., 2017; Markowicz et al., 2018; Rupprecht et al., 2018). The pooled CSF CXCL-13 sensitivity ranged from 89 to 97% and its pooled specificity was 96% (CI_95%_ = 92–98%) in two meta-analyses (961 patients presenting Lyme neuroborreliosis and 3,282 controls) (cf. Figure 1) (Yang et al., 2017; Rupprecht et al., 2018). Other new tools such as CSF fluid free light chain detection (κ, λ FLC, total IgM and albumin) have recently been developed, but they seem to perform worse than CXCL-13 with higher positivity rates in patients with inflammatory neurological diseases, leading to lower specificity (Hegen et al., 2018; Tjernberg et al., 2019).

However, cut-off determination is still an important issue for CSF CXCL-13 detection, since each laboratory had currently selected its own cut-off. The interpretation of the results may, therefore, vary from one laboratory to another (Yang et al., 2017; Markowicz et al., 2018; Pietikäinen et al., 2018; Rupprecht et al., 2018). In addition, no official recommendations have yet determined a threshold. A second issue is the possible elevation of CSF CXCL-13 in other central nervous system diseases (e.g., neurosyphilis, viral meningitides, cryptococcosis, central nervous system lymphoma etc.) (Markowicz et al., 2018; Rupprecht et al., 2018), so these diseases should be screened for in the clinical evaluation of the patients. The third issue is that although CXCL-13 appears to be a good alternative diagnostic test for the diagnosis of early Lyme neuroborreliosis, it has yet to be assessed in late Lyme neuroborreliosis.

#### Modified Two-Tier Testing

Some recent studies suggest that a second-step ELISA may replace the WB in a modified two-tier testing (MTTT) algorithm with equivalent or better accuracy (in particular at the early stage) than conventional two-tier methodology (Branda et al., 2011, 2017; Porwancher et al., 2011; Molins et al., 2016; Lipsett et al., 2019). The MTTT algorithm could combine first-tier testing using a WCS-based ELISA and a second-tier testing with a purified protein ELISA: recombinant VlsE and/or synthetic C6 or pepC10. One study showed an equivalent sensitivity for the diagnosis of European Lyme borreliosis cases compared to a traditional two-tier method, using a WCS-based ELISA as a first step and a C6-ELISA for positive or equivocal results. This protocol also had an equivalent specificity (Branda et al., 2013). More recently, a new type of MTTT algorithm was evaluated using a first-tier recombinant VlsE based-CLIA and then a C6-based ELISA as a second-tier test, with accuracy which was equivalent to a standard two-tier testing (Branda et al., 2017). The FDA has recently validated the commercialization of tests in a modified two-tier methodology using a purified antigen-based ELISA (ZEUS® ELISA Borrelia VlsE1/pepC10 IgG/IgM) followed by a WCS-based ELISA (ZEUS® ELISA *Borrelia burgdorferi* IgG/IgM or IgM and IgG, separately). The CDC recently updated their recommendations for serological diagnosis of Lyme disease along the same lines (Mead, 2019). However, in this context, the role of IB should be discussed as it may remain useful in case of ambiguous or conflicting results.

## Conclusion

In the field of studies on relapsing fever agents, serology plays a role in seroprevalence estimations for emerging agents such as *B. miyamotoi*. For Lyme borreliosis, although serological tools are not perfect when it comes to issues regarding early seronegative window, persistence of antibodies and cross reactions, they remain at the basis of the biological diagnosis of Lyme disease. Consequently, there is a need of continuous improvement of methods (use of recombinant antigens, genospecies diversity, automation, CSF analysis,…) contributing to enhancing the relevance of serological tests for *B. burgdorferi* sl. Also, to avoid misleading interpretations, all international guidelines insist on the need to interpret the results in the light of clinical presentation and duration of symptoms. Better immunological biomarkers in CSF such as CXC-L13 may represent real progress in the diagnosis of neuroborreliosis and new two-tier testing methods will need to be evaluated further in real-life conditions.

## Author Contributions

ET-R and AR provided a first draft of the manuscript. PZ reviewed the manuscript. BJ and CE designed the project of the manuscript and reviewed the final version. CE synthesized the different parts of the manuscript and references.

## Conflict of Interest

The authors declare that the research was conducted in the absence of any commercial or financial relationships that could be construed as a potential conflict of interest.
